# Monoclonal antibody biosimilars for cancer treatment

**DOI:** 10.1016/j.isci.2024.110115

**Published:** 2024-05-24

**Authors:** Linda N. Broer, Daan G. Knapen, Derk-Jan A. de Groot, Peter G.M. Mol, Jos G.W. Kosterink, Elisabeth G.E. de Vries, Marjolijn N. Lub-de Hooge

**Affiliations:** 1 Department of Medical Oncology, University Medical Center Groningen, University of Groningen, Groningen, the Netherlands; 2 Department of Clinical Pharmacy and Pharmacology, University Medical Center Groningen, University of Groningen, Groningen, the Netherlands; 3 Department of Pharmaco-, Therapy-, Epidemiology- and Economy, Groningen Research Institute for Pharmacy, University of Groningen, Groningen, the Netherlands; 4 Department of Nuclear Medicine and Molecular Imaging, University Medical Center Groningen, University of Groningen, Groningen, the Netherlands

**Keywords:** Health sciences, Biological sciences, Immunology, Cancer

## Abstract

Monoclonal antibodies are important cancer medicines. The European Medicines Agency (EMA) approved 48 and the Food and Drug Administration (FDA) 56 anticancer monoclonal antibody-based therapies. Their high prices burden healthcare systems and hamper global drug access. Biosimilars could retain costs and expand the availability of monoclonal antibodies. In Europe, five rituximab biosimilars, six trastuzumab biosimilars, and eight bevacizumab biosimilars are available as anti-cancer drugs. To gain insight into the biosimilar landscape for cancer treatment, we performed a literature search and analysis. In this review, we summarize cancer monoclonal antibodies’ properties crucial for the desired pharmacology and point out sources of variability. The analytical assessment of all EMA-approved bevacizumab biosimilars is highlighted to illustrate this variability. The global landscape of investigational and approved biosimilars is mapped, and the challenges for access to cancer biosimilars are identified.

## Graphical abstract

**Figure:**
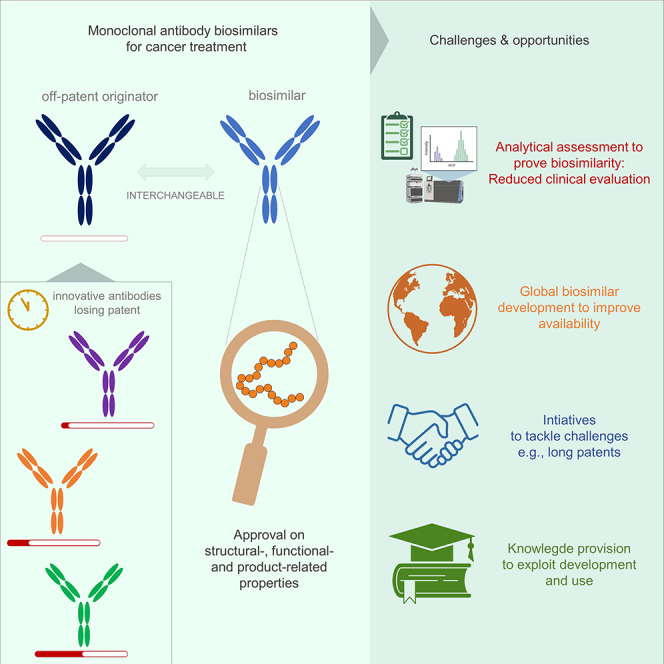

## Introduction

**Figure 1:**
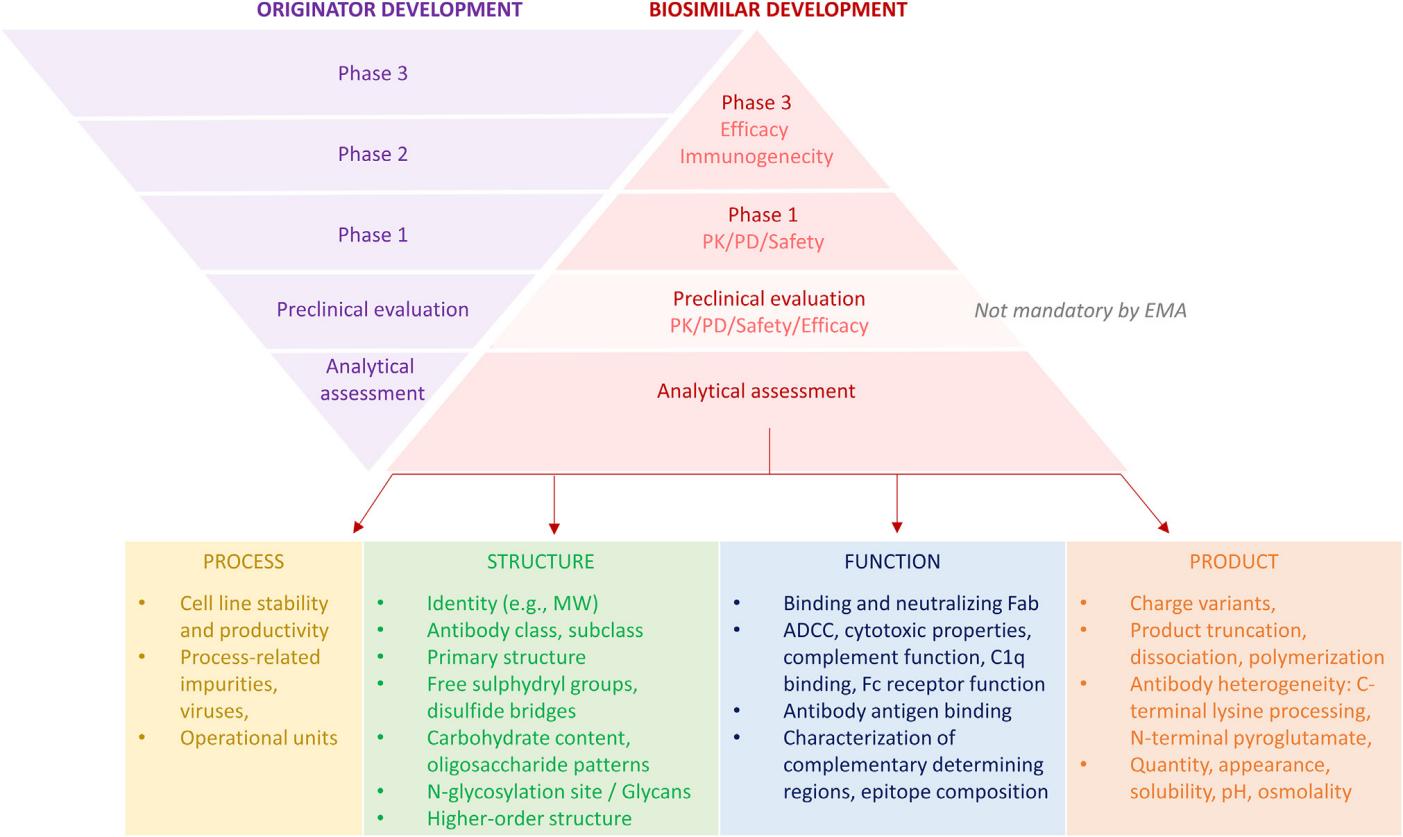
Development phases biosimilar versus originator After analytical characterization and non-clinical studies, originator approval relies on proof of clinical benefit vs. standard care. For biosimilars, the development phases’ importance is opposite to the originator’s: analytical assessment provides the strongest proof of similarity. In humans, a pharmacokinetic study has to be performed, and efficacy is evaluated in one main indication. EMA’s guideline^13^ on monoclonal antibody development describes parameters that need to be controlled regarding process, e.g., cell line stability, continuous capability to produce the desired product quality; impurities, viruses, function operational units, e.g., validation purification column, aseptic filling, column loads, pH, and temperature. Parameters regarding structure are identity, molecular weight, isoform pattern, extinction coefficient, electrophoretic profiles, chromatographic and spectroscopic profiles, antibody class, subclass, light-chain composition, primary structure e.g., peptide mapping, amino acid sequencing, and mass spectrometry analysis, N- and C-terminal amino acids e.g., C-terminal lysine(s), free sulfydryl groups, disulfide bridge integrity/mismatch, carbohydrate content, oligosaccharide pattern (neutral sugars, amino sugars, and sialic acids), N-glycosylation on heavy chains, other glycosylation site(s), glycan structures, mannosylation, galactosylation, fucosylation, sialylation, main glycan structure distribution (e.g., G0, G1, and G2). Regarding function, tests should reflect the clinic e.g., binding and neutralizing. Effector functions (also when not part of mechanism): ADCC, cytotoxic properties, complement binding and activation, C1q binding, Fc gamma- and neonatal receptor binding (cell-based assays preferred); antibody antigen affinity, avidity, and immunoreactivity; crossreactivity with immunohistochemistry; complementary determining regions; target epitope, e.g., protein, oligosaccharide, glycoprotein, glycolipid, amino acid sequence, and carbohydrate structure. Parameters regarding product are charge variants (quantitatively and qualitatively); chromatography/electrophoresis to detect truncation, dissociation, and polymerization, impurities: protein A, host cell proteins, DNA, culture or purification residues, downstream residues; C-terminal lysine processing, N-terminal pyroglutamate, deamidation, oxidation, isomerization, fragmentation, disulfide bond mismatch, N-linked oligosaccharide, and glycation (orthogonal methods). General tests involve drug quantity, appearance, solubility, pH, osmolality, extractable volume, sterility, bacterial endotoxins, and visible and sub-visible particulate matter on batch release and for stability.

Monoclonal antibodies are important cancer medicines. There are 48 approved by the European Medicines Agency (EMA) and 56 by the Food and Drug Administration (FDA), and these numbers will grow.^1^ Their global market burdens healthcare systems and hampers drug accessibility, with low- and middle-income countries having scarce to zero access.^2^^,^^3^ Biosimilars can potentially retain costs and expand drug availability.^4^ Monoclonal antibodies are complex macromolecules manufactured in living cells inherent to molecular heterogeneity, making it impossible to produce an exact copy. A biosimilar is, therefore, by definition, not identical but highly similar to an already-approved off-patent antibody, referred to as the originator.^5^^,^^6^^,^^7^ Unlike a chemically synthesized generic, approved upon a single bioequivalence study, biosimilar approval is based on “the totality of evidence” in a 3-layer similarity comparison with the originator.^8^ The upside-down triangle in Figure 1 emphasizes the analytical assessment in the first layer of biosimilar development, providing the most substantial evidence of similarity because even small differences are detected analytically. In the second layer, similarity of a biosimilar with the originator is assessed in preclinical models, although not required by EMA. The third and smallest layer consists of clinical studies, a phase 1 trial for pharmacokinetics and a phase 3 trial to confirm efficacy and safety for a sensitive indication in a homogenous population to detect the slightest differences with the originator.^8^ So far, EMA approved five anti-CD20 rituximab, seven anti-human epidermal growth factor receptor 2 (HER-2) trastuzumab, and eight anti-vascular endothelial growth factor (VEGF) bevacizumab anti-cancer biosimilars; FDA approved, respectively, three, five, and four.^9^^,^^10^ Despite the urgency, the availability and uptake of anti-cancer biosimilars are divergent among European countries.^11^^,^^12^

In this review, we outline current developments in the complex biosimilar field. In addition to existing reviews from a more regulatory perspective,^14^^,^^15^ we first aim to provide background information on pharmacology of monoclonal antibody biosimilars for cancer, to increase understanding of potential variation and consequences of variation for safety, pharmacokinetics, and efficacy. To demonstrate the reliability of analytical assessments, we provide an overview of EMA-approved bevacizumab biosimilars as a case study. It demonstrates how elaborate and powerful the analytical assessment is, how low variation in practice is, and how the smallest variability is detected, that will not be picked up by large efficacy studies. Hereafter, we mapped available biosimilars worldwide and identified the challenges and opportunities for antibody biosimilar uptake in oncology.

## Search strategy

Relevant English-written articles published until November 2023 were searched in PubMed. Papers concerning cancer monoclonal antibodies and variability were searched using the terms “antibody,” “pharmacology,” “critical quality attribute,” “variability,” or synonyms. The terms “biosimilar” and “cancer” were used to extract ongoing themes that could be identified as challenges for cancer monoclonal antibody biosimilars. From European Public Assessment Reports (EPARs), data regarding analytical assessment of the different approved bevacizumab biosimilars were summarized. Websites www.clinicialtrials.gov and www.gabionline.net were used to identify approved and investigational biosimilars for cancer indications. Additional biosimilars were found on PubMed, websites of pharmaceutical industries, and summaries of global market reports. The websites www.antibodysociety.org, and www.iqvia.com were used to retrieve further relevant information regarding (biosimilar) monoclonal antibodies. Regulatory information was searched on www.who.int, www.ema.eu, and www.fda.gov. The definitions pertinent to this review are detailed in Table 1.

**Table 1:** Definitions

Analytical assessment:	Generation of quality, analytical, and functional data of a drug^5^
Anti-drug antibody formation:	An unwanted immune response against a therapeutic monoclonal antibody^16^
Biological:	A medicinal product whose active substance is made by or derived from a living organism^5^
Biosimilar:	The biological medicinal product, is highly similar to an already authorized biological medicinal product^5^
Cost-effectiveness:	Providing an extra year of healthy life for less than three times the Gross Domestic Product^17^
Critical quality attribute:	Physical, chemical, biological, or microbiological property or characteristic that should be within an appropriate limit, range, or distribution to ensure the desired product quality^18^
Divergence:	When a monoclonal antibody drifts or evolves^19^
Drift:	Unintended or unknown change in the manufacturing process of a monoclonal antibody^19^
European public assessment report:	A set of publicly available documents from EMA, with the complete developmental evaluation, product information, and medicine performance^20^
Evolution:	Deliberate changes in the manufacturing process for product improvement^19^
Extrapolation of indication:	The regulatory and scientific process of granting a clinical indication to a biosimilar extrapolated from one therapeutic indication, relying on the same mechanism of action, not requiring its own efficacy data^5^^,^^21^
Generic:	Chemically synthesized compounds with a simple, well-defined structure independent of the manufacturing process are easy to characterize completely^8^
Immunogenicity:	The extent to which the host’s immune system recognizes and reacts to a monoclonal antibody^22^
Interchangeability:	Refers to exchanging originators with their respective biosimilar, but also exchanging biosimilars that refer to the same originator product^8^
Low- and middle-income countries:	Economies with respectively $1,035 or less and between $1,036 and $12,535 gross national income per capita^23^
Monoclonal antibody:	An antibody derived from the clone of a single B cell produced in large quantities of identical cells possessing an affinity for the same epitope on a specific antigen, e.g., cancer cell^14^
Originator:	Innovative biological developed and patented by a pharmaceutical company^5^
Pharmacology:	Origin, chemistry, and uses of drugs and their effects on the body^24^
Substitution:	Automatically interchanging drugs at the pharmacy level^5^
Switching:	Interchanging originator and biosimilar or between biosimilars^5^
WHO Essential Medicines List:	Essential medicines that satisfy the priority healthcare needs of a population, selected for disease prevalence and public health relevance, evidence of efficacy and safety, and comparative cost-effectiveness. They are intended to be available in functioning health systems at all times^25^

## Pharmacology

**Figure 2:**
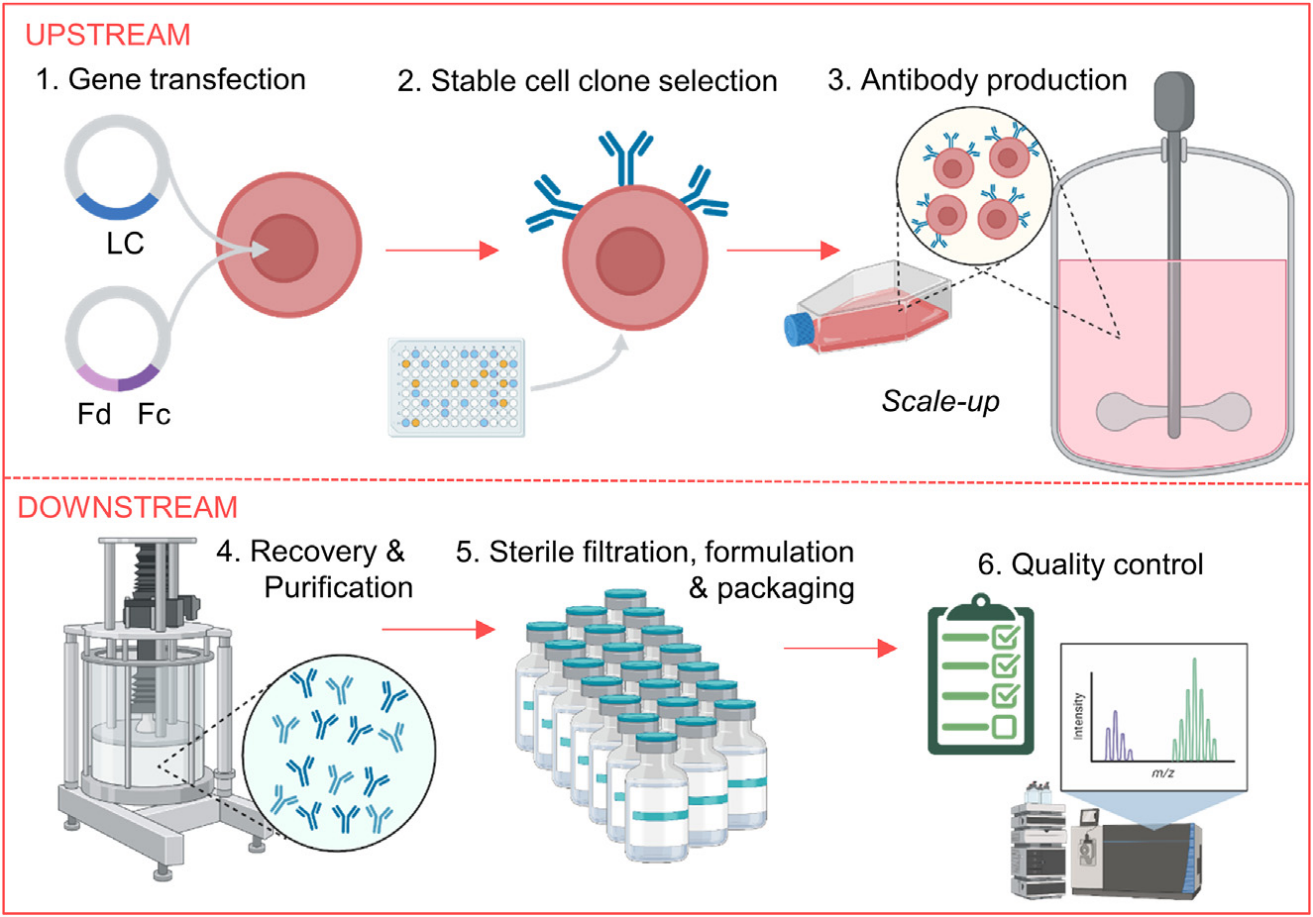
Monoclonal antibody manufacturing Divided into upstream processing, involving gene transfection, stable cell clone selection, and antibody production from mammalian cells on small and large scales, and downstream processing, in which the antibody is recovered and purified through a combination of several methods. The product is then formulated, sterility filtrated, and packaged, followed by final release quality control (Created with biorender.com). LC, light chain; Fc, crystallizable fragment, Fd, heavy chain of the Fab region. With recombinant DNA technique, a vector with genes encoding for the variable and constant region is inserted into host cells, the “expression system,” that will produce the antibody for canonical antibodies, often Chinese hamster ovary or murine lymphoid cells. Expression systems have unique post-translational modifications: glycosylation, phosphorylation, deamidation, methylation, and acetylation, resulting in micro-heterogeneity, even between antibodies from the same cell line.^26^ Smaller constructs, lacking the highly variable Fc glycosylation, can be simpler produced in *Escherichia coli* bacteria.^27^ Immunoconjugates are more complex, with linker and toxin chemistry.^28^^,^^29^ Factors of influence are gene mutations in the host cell DNA, host cell impurities, cell productivity, and protein degradation, potentially leading to aggregates, fragments, unusual glycosylation forms, and charge-heterogeneity. Process parameters such as pH, pressure, temperature, and oxygen supply can also impact product quality.^30^ After antibody production in large bioreactors, isolation and purification steps remove cell-related impurities (host cell DNA, proteins), process-related impurities (buffers), and product-related impurities (aggregates and fragments). Finally, the monoclonal antibody is formulated, sterility filtrated, and packaged. Formulation buffers and storage conditions are critical for the protein’s stability over time.^31^

Their complex structure and manufacture determine the pharmacology of monoclonal antibodies. Figure 2 shows monoclonal antibody manufacturing and sources of variability. Here, we summarize key components of their structural, functional, and product-related aspects and variability that could influence their pharmacological properties.

### Structure

**Figure 3:**
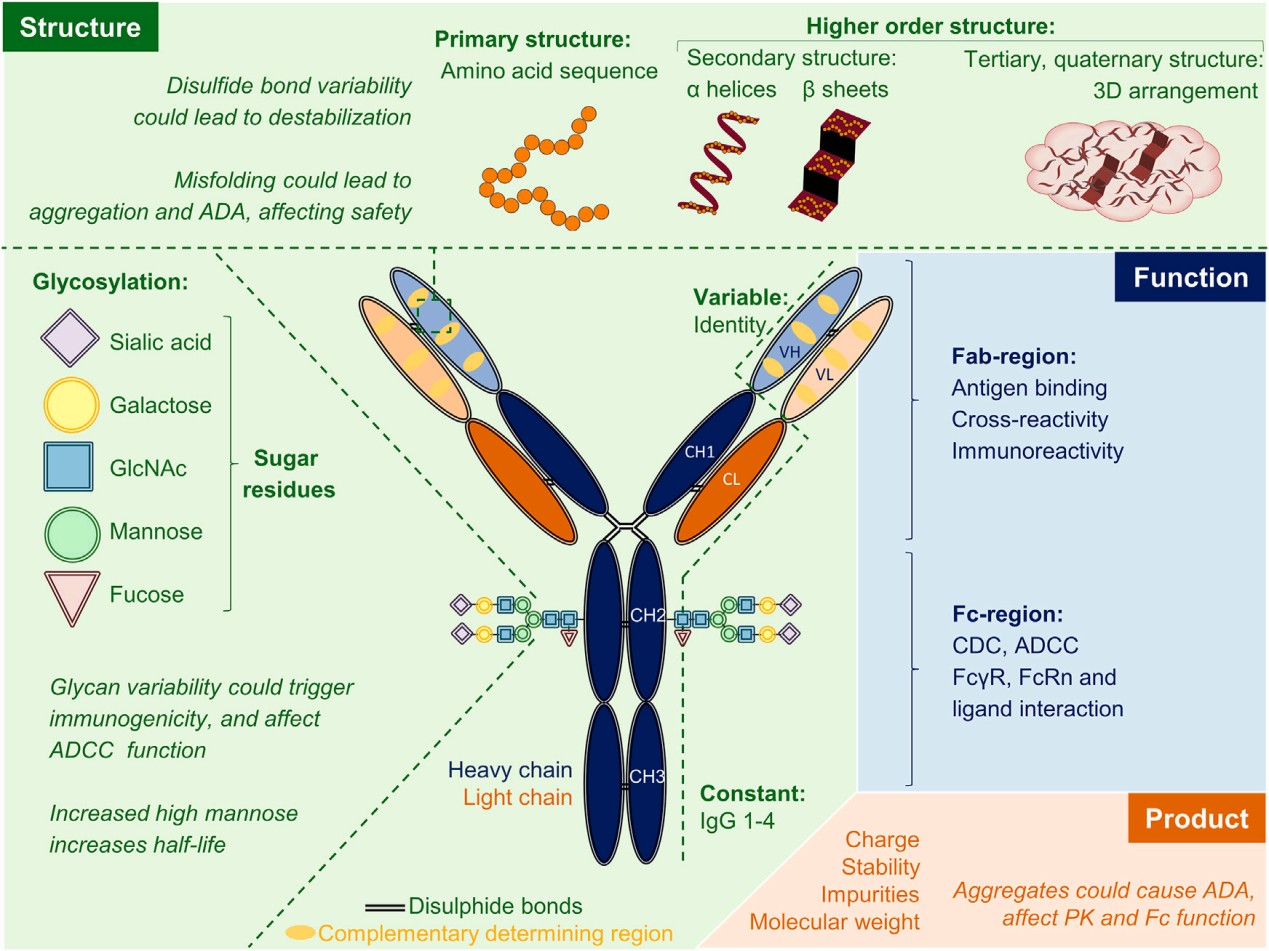
Monoclonal antibody structure, function, and product-related properties Structural properties are in green, functional properties in blue, and product-related properties in orange. Potential effects when changed are indicated in italics. 3D, three-dimensional; IgG, immunoglobulin G; CDC, complement-dependent cytotoxicity; ADCC, antibody-dependent cellular cytotoxicity; Fab, fragment antigen binding; Fc, crystallizable fragment; FcγR, crystallizable fragment gamma receptor; FcRn, crystallizable fragment neonatal receptor; VH, variable heavy chain; CH, constant heavy chain (sub-chains 1–3); VL, variable light chain; CL, constant light chain; PK, pharmacokinetics; ADA, anti-drug antibodies; MW, molecular weight.

Figure 3 shows the typical Y-shape of the monoclonal antibody molecule, consisting of heavy and light chains subdivided into a variable and constant region. The variable region is antibody specific, whereas the constant region is most often the immunoglobulin backbone subtypes 1 or 4.^32^^,^^33^ The variable fragment antigen-binding (Fab) region and the constant fragment crystallizable (Fc) region are responsible for functional properties, e.g., mechanism of action, effector function, and pharmacokinetics. The primary structure of a monoclonal antibody is the amino acid sequence, often humanized or fully human, but older constructs are chimeric (e.g., rituximab and cetuximab). Higher-order structures define the three-dimensional shape. The complementarity-determining region as part of the variable region enables antigen specificity. Disulfide bonds connect all regions for stability. The sugar groups at the constant heavy two domains of the Fc part, called the N-glycans, are important structures because of their strong influence on Fc-function. Novel antibodies are often glycoengineered at this site to influence half-life or effector functions.^34^^,^^35^

### Mechanism of action

Monoclonal antibodies can have different mechanisms of action. In short, monoclonal antibodies can directly target tumor cells by interfering with cell signaling or delivering a toxic payload such as antibody-drug conjugates.^32^^,^^36^ Another major class of monoclonal antibodies exerts immune-mediated tumor cell killing, such as immune checkpoint inhibitors, blocking the programmed death (ligand) 1 (PD-(L)1) axis.^37^ Immune-mediated tumor cell killing can also be caused by Fc-Fcγ receptor interaction with macrophages or natural killer cells. This effect is called antibody-dependent cellular cytotoxicity (ADCC) and complement-dependent cytotoxicity (CDC).^36^ Lastly, monoclonal antibodies can influence the tumor microenvironment, such as vasculature, stroma, or soluble targets, e.g., when targeting VEGF with bevacizumab.^32^^,^^36^

### Pharmacokinetics

The pharmacokinetics of monoclonal antibodies is characterized by a fast distribution over large spaces, such as vasculature, due to their size and polarity, followed by slow elimination, with an elimination half-life of 11–30 days. Monoclonal antibodies have a unique target-mediated clearance via antigen binding, resulting in nonlinear pharmacokinetics at low doses. Most antibodies can be internalized after receptor binding and are then degraded via lysosomes, influenced by their dose, antigen affinity and density, and internalization rate. Non-specific monoclonal antibody clearance is the degradation in off-target cells, the liver, and the mononuclear phagocyte system via Fcγ receptor binding. Interaction with the Fc neonatal receptor extends the half-life by reducing lysosomal degradation in endothelial and bone-marrow-derived cells.^38^^,^^39^^,^^40^

### Safety

Apart from those related to their target, general safety concerns are infusion reactions and the extent of immunogenicity. The latter could lead to anti-drug antibody formation. This can result in reduced efficacy due to target neutralization and accelerated clearance. Human and humanized antibodies are better tolerated than chimeric or murine constructs due to the lower percentage of foreign parts.^41^ The degree of immunogenicity depends on drug dose, administration route, drug-related impurities, aggregates, and variable amino acid sequence or glycosylation and is influenced by the formulation.^16^

The aforementioned structural-, functional-, and product-related properties of monoclonal antibodies are so-called critical quality attributes. When changed, this variability could affect the mechanism of action, pharmacokinetics, and safety. This is summarized in Figure 3.

### Variability

Manufactured monoclonal antibody batches, either originator or biosimilar, display inherently intermolecular heterogeneity, particularly in post-translational modifications, such as glycosylation. Moreover, manufacturing sites and processes are continuously subject to changes to improve production, increase production scale, or transfer to additional production sites.^22^^,^^42^ All these changes may affect quality attributes, potentially leading to changed pharmacology. Unwanted changes, so-called drifts, are rare, with only two reports for monoclonal antibodies used in oncology. One reported drift involved cetuximab, which inhibits epidermal growth factor receptor activation, first approved for colorectal cancer in 2004 in the European Union (EU) and the US in 2011. The cetuximab manufactured in the US showed a 22% higher drug exposure in patients due to decreased clearance than the EU-produced product.^43^ However, post-marketing comparison clinical trials revealed no difference in efficacy and safety.^44^^,^^45^ Another drift case involved the ADCC function of trastuzumab. ADCC is part of trastuzumab’s mechanism of action.^46^^,^^47^ In several of the 203 trastuzumab originator batches, expiring between 2018 and 2019, two drifts were detected in the N-glycans’ sugar residues. In the first case, decreased percentage of afucose caused decreased ADCC and FcγRIIIa binding. In a second drift case, an increased percentage of the high mannose sugar group caused increased ADCC activity and FcγRIIIa binding.^47^ A 3-year follow-up study revealed improved event-free survival of a biosimilar compared to the originator.^48^ Therefore, analysis of N-glycans’ afucose and high mannose is crucial in the analytical assessment of trastuzumab originator and biosimilars.^49^ Besides drifts, monoclonal antibodies are prone to form aggregates, potentially leading to adverse events, such as liver toxicity and immunogenicity in patients.^48^^,^^49^^,^^50^ The host cell and purification processes can influence the heterogeneity of monoclonal antibodies and thus potentially impact pharmacokinetics.^51^ Positively charged antibody variants are cleared faster nonspecifically than less positively charged variants (Figure 3).^38^^,^^40^ Analytical assessment continues during the drug’s lifetime to verify that the monoclonal antibody remains similar.^52^ An extensive set of analytical methods evolved over three decades of protein manufacturing to detect the smallest variations.^22^^,^^53^^,^^54^ Therefore, the analytical assessment enables better distinction in potential differences than pharmacokinetics and efficacy studies in patients. For details, see Figure 1.

## Bevacizumab biosimilars

Each biosimilar application is carefully reviewed by EMA and approved based on a rigorous dataset, including human data for pharmacokinetics, safety, immunogenicity, and efficacy. We used EPARs of all EMA-approved bevacizumab biosimilars to compile an overview of the variability in structural aspects and their extent in practice in the biosimilars compared with their originator Avastin.^55^^,^^56^^,^^57^^,^^58^^,^^59^^,^^60^^,^^61^^,^^62^ During the comparative phase 1 and phase 3 clinical trials of the bevacizumab biosimilars, there were no differences and therefore no concerns, with regard to pharmacokinetics, safety, and efficacy.

Bevacizumab originator was approved by EMA in 2005 to treat patients with colorectal cancer and has been off-patent since 2022. With three additional biosimilars approved in 2022, bevacizumab counts with eight, the most biosimilars currently in Europe. For the details of the analytical assessment of all bevacizumab biosimilars, see Tables 2 and 3. EMA guidelines do not dictate specifically how and to what extent the analytical data should be provided, but the critical quality attributes that impact safety or efficacy should be extensively represented (see also Figure 1).^63^ The guidelines for “biosimilar quality data” are, in principle, based on the guideline for “manufacturing changes for biological products.” Each biosimilar applicant should produce several batches of their product to be compared with the originator. A quality profile should be generated based on the analytical data of several clearly identified originator batches.^64^^,^^65^ The originator batches’ variability range determines the biosimilar batches’ specification limits. Quantitative ranges should be established where possible. When parameters are out of range, this should be accompanied by a justification for why this will not impact product quality, safety, or efficacy.^63^ EPARs summarize the total data collected in a redacted format. Although EPARs are structured similarly, they vary because they are the result of varying assessors and negotiation between EMA and the sponsor. Therefore, not only the methods and extent of analysis but also the publicly available data and the way of reporting vary from biosimilar to biosimilar, as is also demonstrated in our overview presented in Table 2.

**Table 2:** Comparison of analytical assessments of EU-approved bevacizumab biosimilars

	ABP215	PF-06439535	SB8 (*n* = 2)	MB02 (*n* = 2)	MYL-1402O	CT-P16
**Structure**						

Primary structure	7/7 tests similar	7/7 tests similar	2/2 tests similar	4/6 tests similar ↓ glycation ∼ N/C terminal	4/4 tests similar	2/3 tests similar ∼ N/C terminal
Higher-order structure	8/8 tests similar	5/5 tests similar	3/3 tests similar	7/8 tests similar ↑ free thiol	7/7 tests similar	1/3 tests similar ∼ free thiol
Glycosylation	8/9 tests similar ∼ high mannose	1/2 tests similar ↑ mannose 5	1 test ↑ high mannose ↓ afucose	5/7 tests similar ↑ galactose ↑ sialic acid	0/3 tests similar ↑ high mannose OR ↓ Ng-HC ↑ sialic acid	1/4 tests similar ∼ glycan profile ns

**Function**						

Fab-function	6/6 tests similar	2/3 tests ∼KD% VEGF WR	4/4 tests similar	7/7 tests similar	8/8 tests similar	8 tests minor differences ns
Fc-function	9/10 tests similar ∼ FcγRIIIb	4/5 tests similar ∼ FcγRIIIa	1/2 tests similar ∼ KD% FcγRIIIa 158F	10/13 tests similar ∼ FcγRI, FcγRIIIa V/F	9/11 tests similar ∼ FcRn, FcγRIIIb WR	

**Product**						

Molecular weight and impurities	7/8 tests similar ↓ HMW	2/5 tests similar ↓ HMW	0/3 tests similar ↑ subvisible particles ↑ HMW	2/5 tests similar ↓ HMW, ↑ HC + LC ↓ NGHC ↓ IgG, ↑ HHL	3/5 tests similar ↓ HMW ↑ %LC ↓ %HL + 2H	7 tests minor differences ns
Charge	3/4 tests similar ∼ acidic-basic variants	4/5 tests similar ∼ acidic-basic variants	↑ acidic-basic variants	1/2 tests similar ∼ basic variants distribution	1/4 tests similar ∼ basic-main variants ↓ hydrophobic variants ↓ Met-434 oxidation	12 tests minor differences ns

Summary of tests and results from all EMA-approved bevacizumab biosimilars ABP215 (Mvasi), PF-06439535 (Zirabev), SB8 (licensed under trade names Aybintio and Onbevzi), MB02 (licensed under trade names Alymsys and Oyavas), MYL-1402O (Abevmy), and CT-P16 (Vegzelma). Parameters are categorized by structural- (green), functional- (blue), and product-related (orange) properties. Similar means within the variability range of the originator. A detailed overview is shown in Table 3. When a parameter was higher compared to bevacizumab originator, this was indicated with ↑, when lower; indicated with ↓, when difference not specified; indicated with ∼. 2H, heavy-heavy fragment; Fab, fragment antigen binding; Fc, crystallizable fragment; HC, heavy chain; HHL, heavy-heavy light fragment; HMW, high molecular weight species; IgG, immunoglobulin G; KD, dissociation constant; LC, light chain; Met, methionine; Ng-HC/NGHC, N-glycosylation heavy chain; ns, not specified; OR, outside range; VEGF, vascular endothelial growth factor; WR, within range.^56^^,^^57^^,^^58^^,^^59^^,^^60^^,^^61^^,^^62^^,^^63^

**Table 3:** Detailed version of Table 2: analytical assessments of bevacizumab biosimilars

Attribute	Parameter	Method	Result
**MYL-1402O – Abevmy**			

Structure			
Primary structure	Primary sequence	Peptide Mapping	Similar
	Intact mass	LC-ESI-MS	Similar
	Reduced mass	LC-ESI-MS	Similar
	Isoelectric point	cIEF	Similar
Higher-order structure	Secondary structure	Far-UV-CD	Similar
	Tertiary structure	Near-UV-CD	Similar
	Secondary structure	FTIR	Similar
	Free cysteine analysis	RP-HPLC-ESI-MS	Similar
	Disulfide bridging		Similar
	Higher-order structure	DSC	Similar
		Intrinsic fluorescence	Similar
Post-translational modification	Ng-HC and p75	CE-SDS (Reduced)	Less Ng-HC outside the quality range and lower p75 levels. No impact on Fc-functions, no impact in Phase 3
	Afucosylation, total high mannose, and -galactose	NP-HPLC	Higher levels of high mannose, no impact on PK, higher galactose and afucosylated species: No impact on Fc-functions, no impact in Phase 3
	total sialic acid	RP-HPLC	Higher levels of total sialic acid. No impact in Phase 3
Function			
Fab-function	VEGF165 binding	ELISA	Similar
	Inhibition of VEGF165	Induced Proliferation HUVEC	Similar
	Inhibition of VEGF121	Induced Proliferation HUVEC	Similar
	Inhibition of VEGF189	Induced Proliferation HUVEC	Similar
	Inhibition of VEGF165	VEGFR-2 phosphorylation HUVEC-cell-based assay	Similar
	VEGF165 binding kinetics	SPR	Similar
	VEGF165 binding kinetics	SPR	Similar
	VEGF121 binding kinetics	SPR	Similar
Fc-function	FcγRIIIa-V158 kinetics	SPR	Similar
	FcγRIIIa-F158 kinetics	SPR	Similar
	FcγRIa kinetics	SPR	Similar
	FcγRIIa-R131 kinetics	SPR	Similar
	FcγRIIa-H131 kinetics	SPR	Similar
	FcγRIIb kinetics	SPR	Similar
	FcγRIIIb kinetics	SPR	Small difference in KD. The difference is within method variability
	C1q binding	C1q binding ELISA	Similar
	FcRn kinetics	Surface Plasmon Resonance	Broader distribution in the kinetic constants within method variability
	ADCC	Cell-based assay	Similar
	CDC	Cell-based assay	Similar
Product			
General	Protein content	UV-280	Similar
Purity	Sub-visible particles	MFI	Similar
	Monomer and aggregates	SEC-HPLC	Lower HMW species
		AUC	Similar
		SEC-MALS	Similar
	Total fragments	CE-SDS (NR)	Slightly higher %LC and lower %HL and %2H. No impact in Phase 3
Charge variants and oxidation	Isoelectric point	cIEF	Similar
	Deamidation, C-terminal lysine	CIEX-HPLC	Difference basic+main peak: carboxypeptidase B treatment removes C-terminal lysine residues and changes distribution. No impact PK/phase 3
	Hydrophobic variants	HIC	Lower content of hydrophobic variants
	Methionine oxidation	RP HPLC ESI-MS	Lower Met-434 oxidation level

**MB02 – Alymsys/Oyavas**			

Structure			
Primary structure	Intact mass	RPLC-UV/MS	similar
	Reduced and de-N-glycosylated (LC + HC)	RPLC-UV/MS	similar
	Glycation (HC and LC)	RPLC-UV/MS	Slightly lower levels for MB02
	Reducing peptide mapping	by RPLCESI-TOF MS/MS	similar
	Reducing peptide mapping	RPLC UV-MS	similar
	N- and C-terminal integrity	Tryptic mapping RPLC UV-MS	Marginal differences
Higher-order structure	Disulfide bridges	Non-reduced peptide mapping	similar
	Free thiols	Ellman’s test	Slightly higher levels
	Secondary structure	CD	similar
	Tertiary structure	Fluorescence	similar
	Higher-order structure	HDX-MS at peptide and intact level	similar
	Epitope mapping	HDX-MS	similar
	Colloidal stability	DLS	similar
	Structural stability	μDSC	similar
Post-translational modification	Oxidation/deamidation/aspartate isomerization	Peptide mapping (LC-ESI MS/MS)	similar
	O-glycosylation	Peptide mapping	similar
	Site of N-glycosylation	Peptide mapping	similar
	Monosaccharide content	GC-MS	Galactose level higher
	Sialic acids content	UHPLC-FLR	Slightly higher sialic acids content for MB02
	Glycosylation assessment	HILIC-UHPLC-FLR, N glycosylation	similar
		LC-MS	similar
Function			
Fab-function	Binding to VEGF-A165	Competitive binding ELISA	similar
	Binding to VEGF-A165	SPR	similar
	Binding to VEGF-A121, -A189, and -A206	ELISA	similar
	VEGF B, C, and D variants and PlGF	BLI	similar
	Antiproliferation bioassay	HUVEC assay	similar
	VEGF neutralization	VEGF blocker reporter assay	similar
	Blockade of KDR signalization pathway	KDR/KDR dimerization bioassay	similar
Fc-function	ADCC and CDC activity	ADCC and CDC bioassays	similar
	Binding to C1	ELISA	similar
	Binding to FcγRI	SPR	Slightly higher relative affinity, similar KD
	Binding to FcγRIIa	SPR	similar
	Binding to FcγRIIb	SPR	similar
	Binding to FcγRIIIa V variant	SPR and AlphaLISA	Differences in relative binding (AlphaLISA)
	Binding to FcγRIIIa F variant	SPR and AlphaLISA	Differences in relative binding (AlphaLISA)
	Binding to macrophage mannose receptor	BLI	similar
	Binding to FcRn	SPR and ELISA	similar
Product			
Charge	Charge variants	CEX HPLC	Slight difference in MB02 basic peak and distribution of charge variants
		cIEF	similar
General test	Extinction coefficient	Amino acid analysis	similar
	Protein content	UV	similar
Purity	Size heterogeneity	SE HPLC	Lower HMW species
		CE SDS R/NR	slightly higher HC + LC, lower NGHC (R), lower IgG, higher HHL levels (NR)
		SDS-PAGE R/NR	similar
	Aggregate assessment	sv-AUC	lower for MB02
		Isothermal DLS	similar

**ABP215 – Mvasi**			

Structure			
Primary structure	Intact molecular mass: Profile		similar
	Intact molecular mass: Molecular weight		similar
	Reduced and deglycosylated molecular mass of HC and LC: Profile		similar
	Reduced and deglycosylated molecular mass of HC and LC: Molecular weight		similar
	Reduced peptide map: Profile		similar
	Reduced peptide map: amino acid sequence		similar
	Non-reduced peptide map: Profile		similar
	Non-reduced peptide map: Disulfide structure		similar
Glycosylation	Glycan map: Profile		similar
	Glycan map: % high mannose		Minor quantitative differences in specific glycans
	Glycan map: % galactosylation		similar
	Glycan map: % afucosylation		similar
	Glycan map: % sialylation		similar
	cIEF: Profile		similar
	cIEF: Isoelectric point		similar
	Extinction coefficient		similar
	Identity by ELISA		similar
Function			
Fab-mediated activity	Binding to VEGF		similar
	Neutralization of VEGF-mediated proliferation in HUVEC (potency)		similar
	On and off bind rates (VEGF)		similar
	Binding to VEGF isoforms		similar
	Inhibition of VEGFR-2 RTK autophosphorylation		similar
	Specificity by VEGFR-2 RTK autophosphorylation		similar
Fc-mediated characterization	Binding to FcRn		similar
	Binding to FcγRIa		similar
	Binding to FcγRIIa (131H)		similar
	Binding to FcγRIIb		similar
	Binding to FcγRIIIa (158V)		similar
	Binding to FcγRIIIa (158F)		similar
	Binding to FcγRIIIb		Slightly higher relative binding activity for ABP215
	Binding to C1q		similar
	Lack of ADCC activity		similar
	Lack of CDC activity		similar
Product			
Product-related substances and impurities	SE-HPLC: Profile		similar
	SE-HPLC: HMW		Lower levels of high molecular weight species
	rCE-SDS: Profile		similar
	rCE-SDS: HC + LC		similar
	rCE-SDS: NGHC		Higher glycan occupancy, lower fragment species
	rCE-SDS: LMW + MMW		similar
	nrCE-SDS: Profile		similar
	nrCE-SDS: Main peak		Minor differences in partially reduced species
	nrCE-SDS: Pre-peaks		similar
	CEX-HPLC: Profile		similar
	CEX-HPLC: Acidic peaks		Slightly lower acidic- and higher basic variants
	CEX-HPLC: Main peak		similar
	CEX-HPLC: Basic peaks		similar
Thermal stability and degradation	50°C Forced degradation		similar
	40°C Stressed stability		similar
	25°C Accelerated stability		similar
General properties	Protein concentration		similar
	Volume		similar
	Osmolality		similar
	pH		similar
	Appearance		similar
	Color		similar
	Clarity		similar
Process-related impurities	HCP- ELISA		similar
	HCP analysis by LC-MS		similar
	Protein A-ELISA		similar
	Residual DNA-qPCR		similar

**PF-06439535 – Zirabev**			

Structure			
Primary structure and PTMs	Identical amino acid sequence	LC/MS/MS bioinformatics peptide mapping/Edman degradation	similar
	Molecular mass and size	Nanoelectrospray ionization MS	similar
	Posttranslational modifications	Nanoelectrospray ionization MS	similar
		LC/MS, Subunit analysis	similar
		LC/MS and LC/UV, mapping Trypsin	similar
Disulfide bonds	State of cysteines and disulfide bonds	Sulfhydryl analysis	similar
		LC/MS - non-reduced mapping Lys-C	similar
Higher-order structures	Secondary structure	Far-UV circular dichroism spectroscopy	similar
		Fourier transform infrared spectroscopy	similar
	Tertiary structure	Near-UV circular dichroism spectroscopy	similar
		Fluorescence spectroscopy	similar
	Thermal stability	Differential scanning calorimetry	similar
N-linked glycan profile	Distribution, structure, composition, glycosidic linkages, sialic acid levels	HILIC/MS	Slightly higher Man5 levels: No impact on PK
		Exoglycosidase digestion/HILIC	similar
Function			
VEGF binding to Fab domain	Range of inhibition of VEGF response and binding	Inhibition of cell growth assay	Slightly lower cell growth inhibition for PF-06439535, relative potency ranges of both compounds overlap
		VEGF165 binding, ELISA	similar
	Binding to other VEGF isoforms	VEGF121, VEGF189, VEGF206, ELISA	similar
ADCC activity	Lack of ADCC activity	PBMC ADCC assay	similar
FcγR binding	Binding FcγRI/IIa/IIb/IIIa/IIIb	SPR	Minor differences in relative KD (% KD) FcγRIIIa 158F
FcRn binding	Range of FcRn binding	SPR	similar
CDC activity	Lack of CDC activity	CDC assay	similar
	Dose-dependent response curves	C1q binding assay	similar
Product			
Charge heterogeneity	Range of acidic species	iCE	Slightly lower acidic + main species, higher basic species for PF-06439535 due to higher proportion with one/two C-terminal lysines in the heavy chain
	Range of basic species	iCE	similar
	Range of main species	iCE	similar
	Identity major/minor charge isoforms	Cation Exchange-HPLC with MS	similar
		Carboxypeptidase B/iCE	similar
Product purity	Range of monomers levels	SE-HPLC	Higher monomer and lower HMMS levels
	Range of HMMS levels		similar
	Range of HC + LC and fragment levels	CGE (reducing)	Higher HC + LC lower fragment levels
	Range of intact IgG levels	CGE (non-reducing)	Higher level of intact IgG
	Banding pattern	SDS-PAGE (total protein + western blot)	similar
Forced degradation	Conditions: high temperature, light exposure, forced deamination	SE-HPLC/iCE/CGE (R + NR)/cell-based assay/UV spectroscopy/LC/MS/peptide mapping trypsin HIAC	similar

**SB8 – Aybintio/Onbevzi**			

Structure			
Primary structure	Amino acid sequence	Reducing peptide mapping MS	similar
	Molecular mass	Mass spectroscopy	similar
	Carbohydrate side chains	HILIC-UPLC	Markedly higher amount of high-mannose in SB8
			Less afucose for SB8
Higher-order structure	Secondary and tertiary structure	CD spectroscopy	similar
		FTIR	similar
		Intrinsic + extrinsic fluorescence	similar
Function			
Biological activity	Antigen (VEGF-A) binding	ELISA	similar
	VEGF-A neutralization	Reporter gene bioassay	similar
	VEGFR Tyr1175 phosphorylation inhibition	time-resolved fluorescence energy transfer	similar
	Inhibition of HUVEC proliferation	Proliferation assay with fluorescent dye activation	similar
	FcγRn binding	SPR	Minor differences in relative KD (% KD) values for FcγRIIIa 158F
	FcγRI/IIa/IIb/IIIa/IIIb binding	SPR	similar
Product			
Purity	Molecular size in solution	SEC-MALLS	Slightly higher estimated MW for the HMW component
		Analytical ultracentrifuge	Differences in f/fo
	Subvisible particles	Microflow imaging	Higher count of subvisible particles except for the ≥25 μm ones
Charge heterogeneity	Charge-related variants	CEX-chromatography	Less main, higher amount of acidic + basic components
		icIEF	Less main, higher amount of acidic components
		Hydrophobic interaction chromatography	Markedly higher amount of “post-main” fractions

μDSC, differential scanning calorimetry; 2H, heavy-heavy fragment; ADCC, antibody-dependent cellular cytotoxicity; (sy-)AUC, analytical ultracentrifugation; BLI, bio-layer interferometry; CD, circular dichroism; CDC, complement-dependent cytotoxicity; CE-SDS, capillary electrophoresis sodium dodecyl sulfate; CGE, capillary gel electrophoresis; cIEF, capillary isoelectric focusing; C(I)EX, cation (ion) exchange; DLS, dynamic light scattering; ELISA, enzyme-linked immunosorbent assay; ESI, electrospray ionization; Fab, antigen binding fragment; Fc, crystallizable fragment; FcR(n), crystallizable fragment (neonatal) receptor; FL(R)D, fluorescence detection; FTIR, Fourier transform infrared; GC, gas chromatography; HC, heavy chain; HCP, host cell protein; HDX, hydrogen-deuterium exchange; HIC, hydrophobic interaction chromatography; HILIC, hydrophilic interaction chromatography; HL, heavy light chain fragment; HMMS, high molecular mass species; HMW, high molecular weight; HPLC, high-performance liquid chromatography; HUVEC, human umbilical vein endothelial cell; iCE, imaged capillary electrophoresis; icIEF, imaged capillary isoelectric focusing; IEC, ion-exchange chromatography; IgG, immunoglobulin; KD, dissociation constant; KDR, kinase insert domain receptor; LC, light chain or liquid chromatography; LMW, low molecular weight; Lys-C, lysin at C-terminal; MALLS, multi angle laser light scattering; Man5, mannose 5; Met-434, methionine-434; MFI, mean fluorescent intensity; MMW, medium molecular weight; MS, mass spectrometry; NGHC, N glycan heavy chain; NR, non-reduced; OD280, optical density at 280 nmr; PBMC, peripheral blood mononuclear cells; PIGF, placenta growth factor; PK, pharmacokinetics; PTM, posttranslational modification; qPCR, quantitative polymer chain reaction; R, reduced; RPLC, reversed phase liquid chromatography; ESI-TOF, electrospray ionization time-of-flight; RTK, receptor tyrosine kinase; SDS-PAGE, sodium dodecyl sulfate polyacrylamide gel electrophoresis; SEC, size exclusion; SKOV3, ovarian cancer cell line; SPR, surface plasmon resonance; Tyr1175, tyrosine 1175; UHPLC, ultra-high-performance liquid chromatography; UPLC, ultra-performance liquid chromatography; UV, ultraviolet; VEGF(R), vascular endothelial growth factor (receptor).^56^^,^^57^^,^^58^^,^^59^^,^^60^^,^^61^^,^^62^^,^^63^

We found that multiple parameters were evaluated for each attribute, reporting several tests per parameter, ranging from 18 to 52 tests per biosimilar. In Table 2, we summarized detected differences for each biosimilar. MB02 and CT-P16 showed minor differences at the N- and C terminal and different amounts of free thiol compared to the originator. For each biosimilar, differences in sugar residues are detected. Increased high mannose for MYL-1402O was out of range. However, this was accepted since no clinical impact was revealed in the registration data. The apparent different relative binding affinity to VEGF of PF-06439535 compared to the originator, an important aspect of bevacizumab’s mode of action, was within the quantitative range. Related to glycan group variability, each biosimilar also poses differences in the Fc effector functions. Several bevacizumab biosimilars have a reduced amount of high-molecular-weight species, leading to a better safety profile than the originator. Each biosimilar also shows variability in acidic-, basic-, or main charge species. However, the reported differences have no clinical impact. For instance, the slightly higher mannose glycan count of ABP215 did not increase serum half-life in patients.^59^ In general, the overview shows a difference in the number of tests. This could be explained by a distinction between critical quality attributes and quality attributes. Variability of the N terminus, for example, is a quality attribute, mandatory to characterize a biosimilar candidate. However, it is not a critical quality attribute, which allows for more loose variability margins. In contrast, the amino acid sequence, a critical quality attribute, needs to be identical. Glycosylation variability could impact safety, pharmacokinetics, and efficacy. It is therefore defined as a critical quality attribute and thoroughly tested. This also applies to all the functional properties, on both the Fc (ADCC and CDC) and the Fab region (antigen-binding). Fc effector function is responsible for ADCC, and CDC functions, and could potentially be affected by variability in glycosylation sites. Therefore, glycosylation as well as ADCC and CDC function undergo in-depth analysis, regardless of their role in the mechanism of action. This is demonstrated in our overview for bevacizumab, a drug that lacks ADCC and CDC function. The absence of the ADCC and CDC function is proven in cell-based assays for each new biosimilar (Tables 2 and 3). Moreover, there is a distinction between orthogonal testing providing multiple answers (mass spectrometry, for example, gives information on peptide mapping and molecular weight) and linear testing providing single answers (e.g., cell-binding assays).^66^^,^^67^ In summary, most data are provided for Fc-function parameters, namely >20% of total data, displaying 80% similarity with the originator. The least data are provided for charge variants, <10%, showing the highest variability with 50% similarity. The highest levels of similarity were observed in primary structure (90%), higher-order structure (91%), and Fab function (96%), in line with the required identical amino acid sequence and mode of action. Glycosylation parameters were 58% similar, and data regarding molecular weight and impurities presented 54% similarity. These calculations do not include function- and product-related parameters of CT-P16 due to a lack of provided information in the EPARs. In conclusion, the data in the EPARs of bevacizumab biosimilars demonstrate a rigorous registration dataset on all clinically relevant attributes.

## Landscape

**Figure 4:**
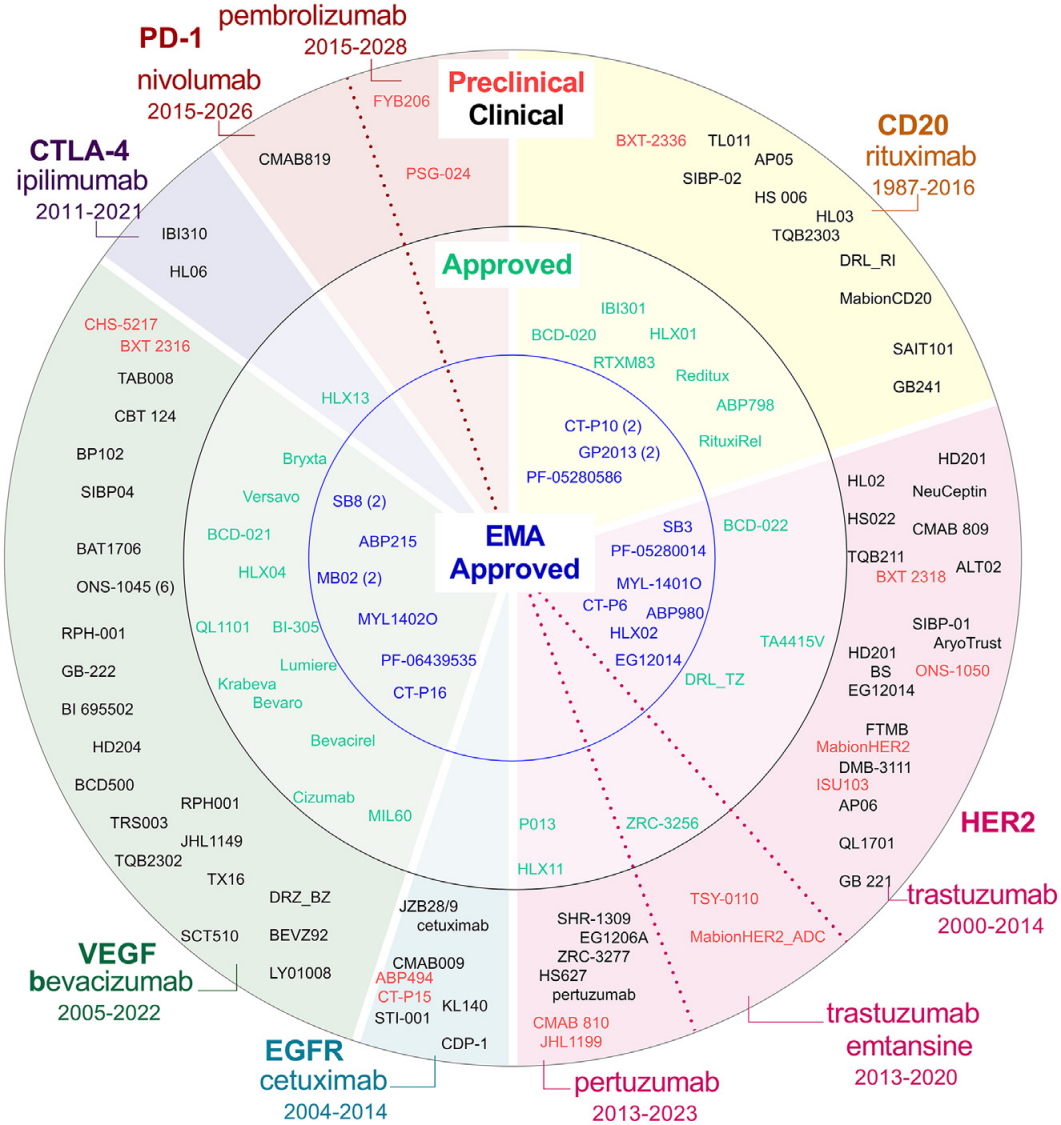
Monoclonal antibody biosimilars in cancer Biosimilars (EMA-)approved, and in (pre-)clinical phase found for cancer indications, patent phase in the European Union indicated per drug. When the same drug is licensed under two trade names, this number is shown in brackets. See Table 4 for details and reference per drug. No biosimilars were found for CD20-targeting ofatumumab and obinutuzumab (off-patent 2023 and 2024), EGFR-targeting panitumumab (off-patent 2018), VEGFR2-targeting ramucirumab (off-patent 2023), or CD30-targeting brentuximab vedotin (off-patent 2021). CD, cluster of differentiation; CTLA-4, cytotoxic T lymphocyte-associated protein 4; EGFR, epidermal growth factor receptor; EMA, European Medicines Agency; HER-2, human epidermal growth factor receptor 2; PD-1, programmed death 1; VEGF(R2), vascular endothelial growth factor (receptor 2). Be aware of the fact that not all countries included in this overview have the same maturity level, based on WHO standards on national regulatory authorities.

Now that it is clear that originators and their biosimilars are clinical equivalents, grounded mainly on analytical data, next, we pursued to map a global biosimilar landscape (Figure 4). The landscape provides an impression of biosimilars under preclinical and clinical evaluation and approved biosimilars, not only by EMA but also outside Europe. This overview is meant as an inventory of what is currently available and what may be expected until 2028. Interestingly, for some monoclonal antibodies, their market exclusivity has expired. Although not all licensed by advanced agencies yet, a few biosimilars have become available, namely for ado-trastuzumab emtansine, cetuximab, panitumumab, brentuximab-vedotin, and ipilimumab. In the EU, even fewer are available, namely for only 3 out of 8 monoclonal antibodies for cancer, with expired patents.^68^ The high amount of biosimilars for trastuzumab and bevacizumab matches the large patient populations that can be treated with monoclonal antibodies and therefore, market size.^69^ The rituximab biosimilar proportion is smaller but significant with indications for autoimmune diseases such as rheumatoid arthritis and included even on World Health Organization’s (WHO) Essential Medicines List for diffuse large B cell lymphoma, chronic lymphocytic leukemia, and follicular lymphoma.^25^ India and China are prominently represented in biosimilar development; however, be aware of the fact that not all countries in this overview have national regulatory authorities with equal WHO maturity levels (Table 4). Interesting phenomena are the PD-1 and PD-L1-targeting monoclonal antibodies that continuously enter the market.^1^ Although labeled innovative, they are rather “me-too” drugs, but not biosimilars. Despite distinct structural differences, they are considered interchangeable.^70^ For instance, pembrolizumab is a humanized antibody, binding with a different affinity to a different PD-1 epitope than the fully human antibody nivolumab, meaning that their structure, especially their complementary determining regions are entirely different, as well as the amino acid sequence of both compounds.^71^ However, they show highly similar clinical efficacy.^70^ Given the upcoming EU patent expirations of nivolumab (2026) and pembrolizumab (2028), there are likely more biosimilars within invisible pipelines than we could find. The development and use of PD-1 antibody biosimilars will have a major clinical and financial impact, considering that nivolumab and pembrolizumab are dominating the global antibody market.^69^ Rituximab, trastuzumab, nivolumab, and pembrolizumab (the last two only for metastatic melanoma) are on WHO’s Essential Medicines List, which makes it paramount that they become globally available and affordable as biosimilars.

**Table 4:** Monoclonal antibody biosimilars in cancer: details of Figure 4

Status	Name	Company	Country	Reference
Rituximab – CD20 antibody				
Preclinical	BXT-2336	BioXpress Therapeutics	Switzerland	^72^
Phase 1	AP05	Aprogen	South Korea	^73^
Phase 3	SIBP-02	Sinopharm	China∗	NCT04361279
	DRL_RI	Dr. Reddy’s Laboratories	India∗	NCT03976102
	HS 006	Hisun Pharmaceutical	China∗	CTR20180855
	HL03	Hualan Biological Engineering	China∗	CTR20190424
	TQB2303	Chia Tai Tianqing	China∗	CTR20182377
	MabionCD20	Mabion	Poland	NCT02617485
	SAIT101	Archigen Biotech	China∗	NCT04361279
	GB241	Nanjing Yoko Pharmaceutical	China∗	NCT03003039
Approved	Reditux	Dr. Reddy’s Laboratories	India∗	^74^
	BCD-020	BIOCAD	Russia	^75^
	RTXM83	mAbxience	Spain	^76^
	RituxiRel	Reliance Life Sciences	India∗	^77^
	HLX01	Henlius	China∗	^78^
	ABP798	Amgen	USA	^79^
	IBI301	Innovent	China∗	^80^
EMA-approved	CT-P10 (X2)	Celltrion Healthcare	South Korea	^81^
	PF-05280586	Pfizer	USA	^81^
	GP2013 (X2)	Sandoz	Europe	^81^
Cetuximab – EGFR antibody				
Preclinical	ABP494	Amgen	US	^82^
	CT-P15	Celltrion Healthcare	South Korea	^82^
Phase 1	JZB28/9	Henlius	China∗	CTR20210716
Phase 3	STI-001	Mabtech	China∗	^82^
	cetuximab	CinnaGen	Iran	NCT03391934
	CMAB009	Mabpharm	China∗	CTR20170701
	KL140	Kelun Pharma	China∗	NCT04835142
	CDP-1	Dragon Boat Pharmaceutical	China∗	NCT03881787
Ipilimumab – CTLA-4 antibody				
Phase 1	IBI310	Innovent	China∗	NCT04868760
	HL06	Hualan Biological	China∗	CTR20190661
Approved	HLX13	Henlius	China∗	^83^
Nivolumab – PD-1 antibody				
Phase 1	CMAB819	Mabpharm Limited	China∗	NCT04659369
Pembrolizumab – PD-1 antibody				
Preclinical	PSG-024	PersisGen Par	Iran	^84^
	FYB206	Formycon	Germany	^85^
Trastuzumab – HER-2 antibody				
Preclinical	BXT-2318	BioXpress Therapeutics	Switzerland	^9^
	MabionHER2	Mabion	Poland	^86^
	ISU103	ISU Abxis	South Korea	^87^
	ONS-1050	Oncobiologics	USA	^88^
Phase 1	NeuCeptin	NeuClone	Australia	ACTRN126-18001657213
	CMAB 809	Mabpharm	China∗	CTR20190897
	ALT02	Alteogen	South Korea	NCT03242239
	SIBP-01	Sinopharm	China∗	^89^
	AryoTrust	AryoGen Pharmed	Iran	^90^
	FTMB	Synthon Chemicals	Netherlands	^91^
	DMB-3111	Meiji Seika Pharma	Japan	^92^
Phase 3	AP06	Aprogen	South Korea	^93^
	QL1701	Qilu Pharmaceutical	China∗	CTR20192189
	GB 221	Genor Biopharma	China∗	NCT04164615
	HL02	Hualan Biological Engineering	China∗	CTR20190665
	HS022	Hisun Pharmaceutical	China∗	CTR20180362
	TQB211	Chia Tai Tianqing	China∗	CTR20181909
	HD201	Prestige Biopharma	EU	NCT03013504
	BS	Pfizer	USA	NCT04181333
	EG12014	EirGenix	China (Taiwan)∗	NCT03433313
Approved	TA4415V	Orchid Chemicals & Pharmaceuticals	Iran	^94^
	DRL_TZ	Dr. Reddy’s Laboratories	India∗	^95^
	Hervycta	Dr Reddy’s Laboratories	India∗	^96^
	BCD-022	BIOCAD	Russia	^97^
EMA-approved	ABP 980	Amgen	US	^81^
	PF-05280014	Pfizer	US	^81^
	HLX02	Accord Healthcare	Germany	^81^
	SB3	Samsung Bioepis	Netherlands	^81^
	MYL-1401O	Viatris	US	^81^
	CT-P6	Celltrion Healthcare	South Korea	^81^
	EG12014	Sandoz GmbH	Austria	^81^
Trastuzumab emtansine – HER-2 ADC				
Preclinical	MabionHER2_ADC	Mabion	Poland	^86^
	TSY-0110	Formosa Pharmaceuticals	China (Taiwan)∗	^98^
Approved	ZRC-3256	Zydus Lifesciences	India∗	^99^
Pertuzumab – HER-2 antibody				
				
Preclinical	CMAB 810	Mabpharm Limited	China∗	^100^
	JHL1199	JHL Biotech	China (Taiwan)∗	^101^
Phase 1	SHR-1309	Jiangsu Hengrui Pharmaceuticals	China∗	^102^
	EG1206A	EirGenix	Germany	NCT05471648
Phase 3	ZRC-3277	Zydus Lifesciences	India∗	NCT05283837
	H S627	Hinsun	China∗	NCT04514419
	pertuzumab	CinnaGen	Iran	NCT04957212
Approved	HLX11	Henlius	China∗	NCT05346224
	P013	Orchid Chemicals & Pharmaceuticals	Iran	^103^
Bevacizumab – VEGF-A antibody				
Preclinical	BXT-2316	BioXpress Therapeutics	Switzerland	^9^
	CHS-5217	Coherus BioSciences	US	^104^
Phase 1	RPH001	R-Pharm	Russia	NCT03659305
	JHL1149	JHL Biotech	China∗	NCT03576651
	TX16	Tanvex BioPharma	China (Taiwan)∗	^104^
	SCT510	Sinocelltech	China∗	NCT05113511
	DRZ_BZ	Dr. Reddy’s Laboratories	India∗	^105^
	BEVZ92	mAbxience	Spain	NCT02069704
	LY01008	Boan Biotech	China∗	NCT05110118
	RPH-001	R-Pharm	Russia	NCT03659305
	GB-222	Genor Biopharma	China∗	NCT04175158
Phase 3	BI 695502	Boehringer Ingelheim	Germany	^106^
	HD204	Prestige Biopharma	Singapore	NCT03390686
	BCD500	BIOCAD	South Korea	^104^
	TRS003	Zhejiang Teruisi Pharmaceutical	China∗	NCT05378867
	TQB2302	Chia Tai Tianqing	China∗	CTR20180857
	TAB008	TOT Biopharm	China∗	NCT05427305
	CBT 124	Cipla	India∗	NCT02879097
	BP102	Jiangsu Hengrui Medicine	China∗	NCT05169801
	SIBP04	Sinopharm	China∗	NCT05318443
	BAT1706	Bio-Thera Solutions	China∗	CTR20170799
	ONS-1045 (6)	Oncobiologics	USA	^104^
Approved	Bryxta	Zydus Lifesciences	India∗	^104^
	Versavo	Dr. Reddy’s Laboratories	India∗	^104^
	BCD-021	BIOCAD	Russia	^107^
	HLX04	Henlius	China∗	^108^
	QL1101	Qilu Pharmaceutical	China∗	CTR20161024
	IBI-305	Innovent	China∗	CTR20160848
	Lumiere	Laboratorio Elea	Argentina	^104^
	Krabeva	Biocon	India∗	^104^
	Bevaro	Zydus Lifesciences	India∗	^104^
	Bevacirel	Reliance Life Sciences	India∗	^104^
	Cizumab	Hetero Labs	India∗	^104^
	MIL60	MAB Works	China∗	^104^
EMA-approved	ABP215	Amgen	Ireland	^81^
	PF-06439535	Pfizer	Europe	^81^
	SB8	Samsung Bioepis	Netherlands	^81^
	MB02	STADA Arzneimittel	Germany	^81^
	MYL-1402O	Mylan	USA	^81^
	CT-P16	Celltrion Healthcare	South Korea	^81^
	SB8	Samsung Bioepis	Netherlands	^81^
	MB02	mAbxience	Spain	^81^

ADC, antibody-drug conjugate; CD, cluster of differentiation; CTLA-4, cytotoxic T lymphocyte-associated protein 4; EGFR, epidermal growth factor receptor; EMA, European Medicines Agency; HER-2, human epidermal growth factor receptor 2; PD-1, programmed death 1; VEGF(R2), vascular endothelial growth factor (receptor 2). Be aware of the fact that not all countries included in this overview have the same maturity level, based on WHO standards on national regulatory authorities. Note: Countries indicated with an ∗ have a maturity level 3 with regard to their regulatory system, according to WHO standards (stable, well-functioning, and integrated). The other countries have a maturity level 4 (Advanced level of performance and continuous improvement).^109^

## Challenges

The themes identified as challenges for cancer monoclonal antibody biosimilars extracted from our search were development costs and drug prices, confirmatory efficacy trials, patents, access in low- and middle-income countries, implementation, and interchangeability.

### Development costs and drug prices

The first hurdle in developing monoclonal antibody biosimilars is their complex and therefore costly manufacturing and extensive analytical assessment.^110^ Development can be improved to some extent by computational tools, *in silico* methods, and innovative high-throughput technologies.^111^ Strikingly, between now and 2030 for less than 50% of exclusivity-expiring molecules, biosimilars are in the pipeline.^12^ It seems that even in rich countries, at this moment, the investment to develop a biosimilar might not be attractive among other reasons due to high demands by regulators.^112^ The majority of development costs are spent on clinical studies. Reducing the mandatory clinical data as currently being explored by regulators might improve prospects.^113^^,^^114^ Also, improvements in analytical testing and modeling alternatives could provide smart solutions and might further reduce costs.^14^ There is great variety in cost-effectiveness among countries, and not a straightforward answer to cost-effectiveness of biosimilars. Apart from the lack of transparency in development costs, this is also due to the differences in healthcare systems. It is therefore difficult to compare different countries. Factors that play a role are for example local, regional, and national health policy; local and central government decisions on reimbursement; and distribution of drugs to hospitals.^15^

Whereas generics can be sold for a fraction of the original drug price, cost savings of biosimilars is divergent in Europe. The modest list price reductions so far show that most robust savings should be in Poland and Germany, with 46% and 40% price reduction, whereas in the United Kingdom and Norway, prices increased with 10% and 5%.^12^^,^^115^ Following savings on antibodies in the rheumatology therapeutic area, we may expect up to 69% price reductions due to biosimilar use.^116^^,^^117^ Several studies predicted favorable cost-effectiveness of monoclonal antibody biosimilars in cancer. However, in order for this to happen on a wider scale, regulations and policies need to be improved, such as more efficient budget allocation, patent assistance programs, flexible willingness-to-pay thresholds, increased biosimilar use, and reduced biosimilar development costs.^118^^,^^119^^,^^120^^,^^121^^,^^122^^,^^123^ At this moment, for the industry, the low biosimilar prices in the competitive market do not warrant biosimilar development at relatively high costs.

### Confirmatory efficacy trials

A thorough overview of how biosimilars are evaluated in clinical phase 1 and 3 studies has been described previously.^124^^,^^125^^,^^126^ In some countries, such as Sweden and the US, additional data on switching from originator to biosimilars are required to allow interchangeability.^127^^,^^128^ Two systemic reviews on biosimilar switching studies, including cancer monoclonal antibodies, show no clinically meaningful differences after switching.^129^^,^^130^ In addition, evidence is building that phase 3 trials might not add additional value for biosimilars.^131^^,^^132^ This creates a discussion in health organizations to adjust the approval pathway. For example, the Medicines and Healthcare Products Regulatory Agency in the United Kingdom stated in May 2021 that confirmatory efficacy trials would no longer be necessary for biosimilars when scientifically justified.^133^ EMA-associated scientists analyzed all approved biosimilars and concluded that in none of the approvals the patient trial played a decisive role.^134^ Recently, the EMA published a summary of analytical data of bevacizumab and adalimumab biosimilars. They confirm our findings regarding our analysis of the EPARs of the bevacizumab biosimilars. They firmly stated that clinical efficacy data were of low relevance regarding quality concerns, urging to redefine the requirements of the clinical evaluation of biosimilars.^21^ In addition, they investigate the suitability of biosimilar development based on quality data, and waiver of clinical efficacy and safety trials on a case-by-case approach reported in their scientific advice from September 2022.^135^ This may result in faster and cheaper availability of biosimilars. The development time for an innovative monoclonal antibody is generally 10–12 years, and for a biosimilar still 8–10 years^136^ During the COVID-19 pandemic, the launch of anti-COVID-19 antibodies happened within several months. The licensing of the product and patent suspension were being discussed by governments.^137^^,^^138^ The same collaboration could support and broaden the availability of cancer monoclonal antibodies and their biosimilars. Overall, it appears that there is a call for standardization, as is proposed in several papers, working toward shorter clinical evaluation and investment in analytical methods to replace patient studies.^139^^,^^140^^,^^141^

### Patents

Originator patent expirations allow for healthy market competition with their biosimilars, which is expected to decrease the global costs of all EMA-approved biosimilars for the 3 reference products, namely rituximab, trastuzumab, and bevacizumab for the targets CD20, HER-2, and VEGF, respectively.^69^ The exponential growth of the monoclonal antibody market over the last 10 years, especially for cancer indications, partially explains the current gap between the high number of marketed originators and the low number of marketed biosimilars. It also indicates that a similar exponential biosimilar market growth might be expected once market exclusivities start lifting further. However, patents for these complex products are long, generally 10 years in the EU and 12 years in the US, with potential extensions.^142^ This time allows the innovator company to cover the high development costs and make profits and drives innovation. Development costs of new medicines are for the major part capital costs, and there is no relation with drug prices and manufacturing costs. However, the current development costs for an innovator company is not in line with what the system pays for a new drug, respectively 200–300 million vs. 2–3 billion US dollars as reported by Strategies in Regulated Markets.^113^ In the EU patent, regulations are overall liberal and different per country. This allows biosimilar developers to wait out key patents in most countries or launch a biosimilar at risk, with no or low punitive damage, in, for instance, the Netherlands.^143^ Long patents are an issue in the US, more than in other countries. Many patents can be applied for, also after market launch, in addition to product and manufacture.^144^ To avoid infringements in the US between originator and biosimilar companies, a so-called “patent dance” is put in place, involving back-and-forth communication between the competing companies.^145^ Pembrolizumab exemplifies an over-patented drug in the US. Of more than 100 patent applications, 53 have been granted. Pembrolizumab’s patent duration is extended with 8 years, with estimated extra drug costs of $137 billion.^146^ Pembrolizumab is expected to predominate the monoclonal antibody market by 2024, with a predicted $18 billion global annual sales.^147^ Patent strategies by pharmaceutical companies to keep a product exclusive are the addition of new indications (e.g., glioblastoma for bevacizumab), a new route of administration (e.g., subcutaneous for trastuzumab), or new drug formulations.^148^ Another opportunity for biosimilar companies is a “skinny label,” which refers to the approval pursuit of a biosimilar for a single indication and not all indications for which the brand name of the drug is approved. Skinny labeling is a measure to create early competition of biosimilars with their originators, paramount, since this will likely lead to substantial cost savings.^149^ Medicines Patent Pool is an initiative, striving through voluntary licensing and patent pooling, to allow valuable medicines to reach low- and middle-income countries.^150^

### Access in low- and middle-income countries

Biosimilars are an entrance to cancer treatment for countries lacking the resources for innovative monoclonal antibodies. There is a communication and knowledge barrier between low- and middle-income countries and supporting agencies, e.g., WHO, that needs to be bridged.^149^ In order to reach low- and middle-income countries, key elements were recently identified, namely, prioritizing targets according to impact on public health, supporting biosimilar development, market-entry, and use, in a country-specific manner.^151^ An overview of biosimilar access in 40 countries based on licensing is given by Huang et al.^152^ Their review showed that Asia has the most biosimilars available, whereas, for Africa, there was only one biosimilar for rituximab at that time. They stated, too, that determining the actual access is far more complex, depending on barriers such as government reimbursement, out-of-pocket costs, budget allocations for biosimilars, shortages, and patent rights.^153^^,^^154^ The authors of these papers state that there is no simple solution to balancing universal guidelines and country-specific needs. Another article describes legal and regulatory issues in such countries, lack of research infrastructure, and educational barriers.^155^ The availability of rituximab biosimilars in India has dramatically improved treatment access, from 35% to 95% of the patients with large B cell lymphoma.^156^ Furthermore, a comparison study between US and India reveals that the treatment of these patients is now similar between these two countries.^157^ Several papers describe the concerns of biosimilar use in Latin America, such as non-adherence to already inconsistent regulations. There is also a need for traceability and pharmacovigilance of biosimilars and precise use of interchangeability. This requires educational efforts in Latin America.^158^ Furthermore, the “biosimilars” on their markets are not rigorously compared with the originator.^159^^,^^160^^,^^161^ A budget impact analysis in 13 countries in the Middle East and North Africa predicted a substantial cost-saving effect of a rituximab biosimilar, assuming a 30% lower drug price.^162^ This indicates the importance of biosimilars in becoming more available in their markets. Several goals are pursued by health organizations such as the American Society of Clinical Oncology (ASCO), European Society of Medical Oncology (ESMO), and WHO, e.g., increasing global access to WHO’s Essential Medicines List, working toward establishing value-based drug pricing, and pricing based on country-specific recourses and cancer burden.^163^^,^^164^ Moreover, between 2019 and 2022, 6 rituximab products and 10 trastuzumab products have been approved via the WHO prequalification program.^165^ This might improve the scarcity of biosimilar global access.^166^^,^^167^^,^^168^^,^^169^^,^^170^

### Implementation and interchangeability

Biosimilar market penetration requires active promotion and financial awareness of stakeholders, e.g., payers, pharmacists, prescribers, and patients.^171^ In Europe, Denmark and the Netherlands switch the most whereas Bulgaria and Belgium performed worst.^12^ Recently, a policy review concluded how EU, US, and Japanese regulations could be improved by addressing region-specific competition barriers and educational needs.^172^ Health organizations, e.g., ASCO, ESMO, and WHO, have already undertaken initiatives to provide biosimilar information and education for healthcare providers and patients.^155^^,^^163^^,^^173^^,^^174^ Real-world evidence of biosimilar interchangeability is building up for rituximab, trastuzumab, and bevacizumab, which can further improve prescriber and patient confidence.^174^^,^^175^^,^^176^^,^^177^ Education surveys reveal the improvements in biosimilar knowledge and acceptance in the US and EU, but they also indicate the importance of the continuous provision of information.^178^^,^^179^^,^^180^^,^^181^^,^^182^^,^^183^ EMA has now released the statement that biosimilars are, upon approval, interchangeable with their originator and with biosimilars referring to the same reference product. This will increase their use.^30^ This will eventually also allow the extrapolation of indication, using biosimilars for off-label indications relying on the proven mechanism of action they have been approved for. There is, however, still a conceptual difference of the meaning of interchangeability as used in the US compared to the rest of the world. In the US, interchangeability is a specific legal status for a biosimilar, awarded by the FDA upon fulfilling considerable additional requirements, such as a multi-switch trial. This may confuse prescribers in the world, suggesting 2 standards for biosimilars.^184^

## Concluding remarks

The current landscape of biosimilars to treat cancer indicates that at the moment, biosimilar development may not be an attractive investment. To fully exploit biosimilar development and use, slimming of the clinical data package might be essential as is already explored by regulators. Much biosimilar development is going on, yet their increased uptake and cost-saving effect can only happen if challenges, described in our review, are tackled. Opportunities for improvement in this highly complex field lay in the pricing, reimbursement, and long patent duration for originator monoclonal antibodies. These issues are being addressed by close interaction between regulators, health technology assessment bodies, and other relevant initiatives, such as Medicines Patent Pool. Lastly, a continuous provision of knowledge and financial awareness is paramount, pursued by ASCO, ESMO, and WHO, which will eventually lead to affordable monoclonal antibody cancer treatments and access to it in all corners of the world.
